# Supplementary material for: Rhythmic glucose metabolism regulates the redox circadian clockwork in human red blood cells
Source: Nat Commun. 2021 Jan 15;12:377. doi: 10.1038/s41467-020-20479-4 (PMC7810875; doi:10.1038/s41467-020-20479-4)
Supplement: Supplementary file 1 — Supplementary Information [file 41467_2020_20479_MOESM1_ESM.docx]

**Supplementary Information**

Supplementary Figure 1


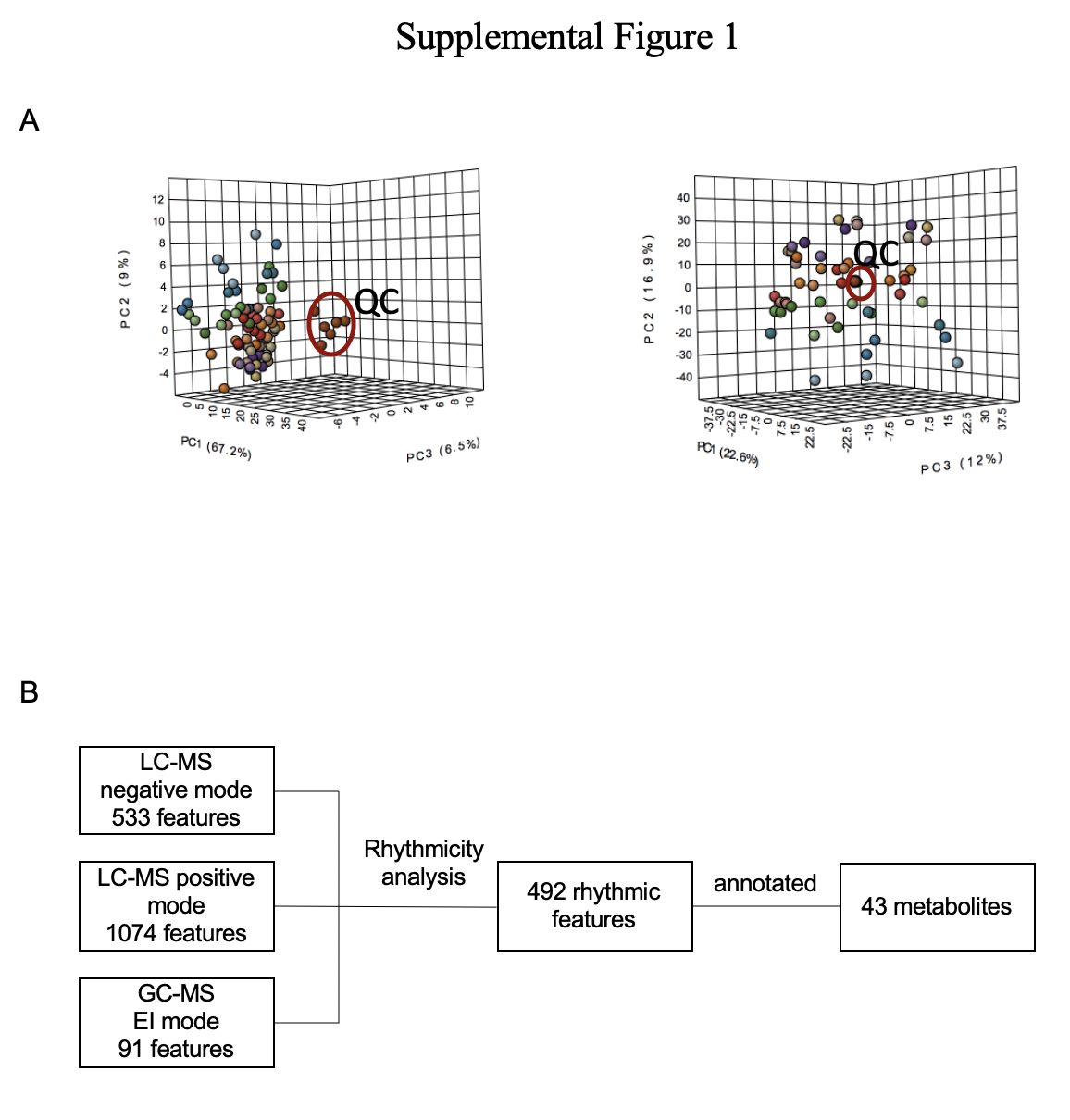


**Supplementary Figure 1. Quality control metrics and analysis of human red blood cell metabolites (Related to Figures 1-2)**

(A) Quality control (QC) data for LC- and GC-MS analyses. Principal components analysis (PCA) was performed on raw feature quantifications. This demonstrated good clustering of pooled biological QC samples, which were run periodically during data acquisition to check for any variance in mass spectrometer performance. (B) Schematic showing the workflow of untargeted metabolomics data analysis.

Supplementary Figure 2


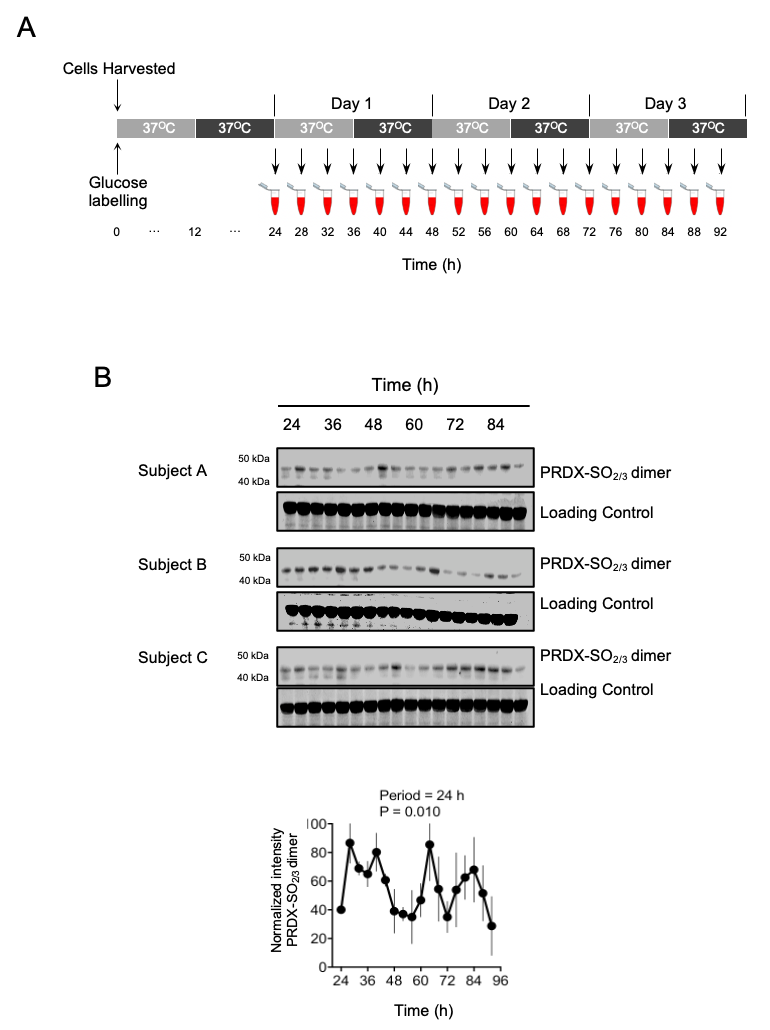


**Supplementary Figure 2. Peroxiredoxin oxidation rhythms in human red blood cells (RBCs) labeled with 2-^13^C_1_-Glucose (Related to Figure 3D)**

(A) Schematic showing experimental protocol used to collect samples. RBCs from n = 3 human subjects were incubated with 11mM 2-^13^C_1_-glucose and kept under constant conditions (37^o^C in continuous darkness) and sampling was performed every 4 hours after reaching steady state (see main text for details). (B) Immunoblots showing rhythms in oxidation of peroxiredoxin (PRDX-SO_2/3_) dimer with loading controls (Coomassie blue gel images). These blots correspond to flux labeling experiments shown in Fig 3B-C. Quantification of blots (mean ± s.e.m.) by densitometry is shown below (n = 3 biological replicates). Values are normalized to the maximum density within each blot. Uncropped blot images are shown in fig. S3. P values were obtained from rhythmicity analysis using RAIN algorithm

Supplementary Figure 3


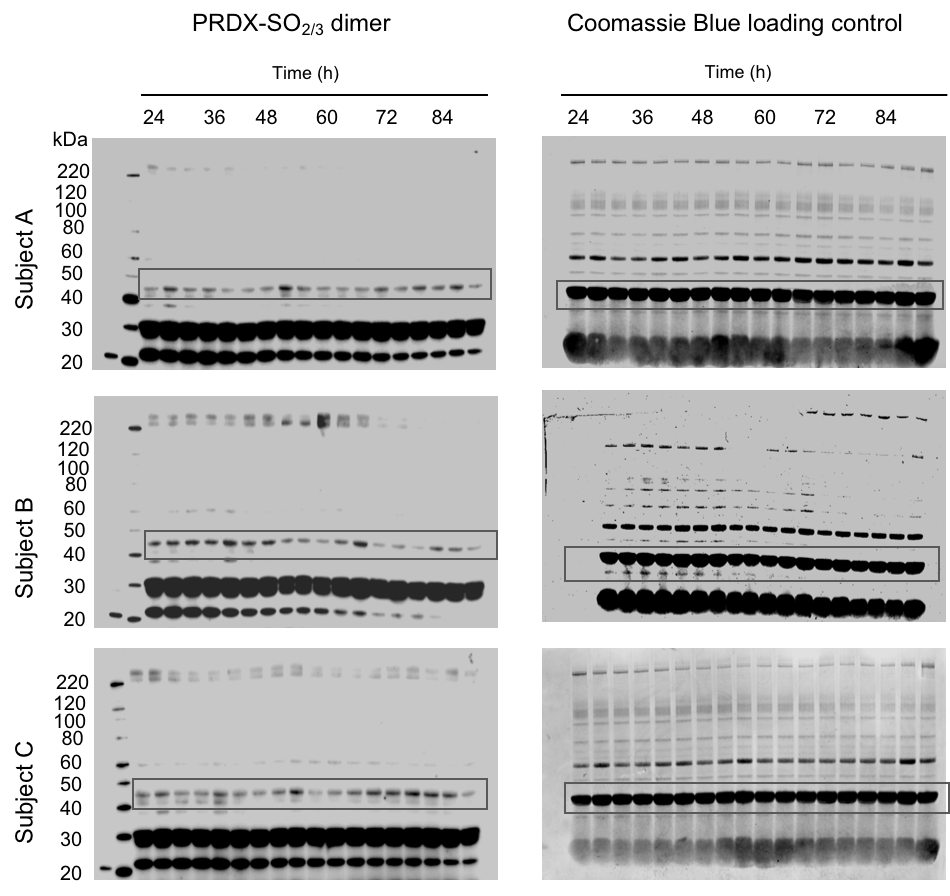


**Supplementary Figure 3. Circadian rhythms in oxidation of peroxiredoxin (PRDX-SO_2/3_ dimer) in human red blood cells (RBCs) (Related to Figure S2 and Figure 3D)**

Images of whole immunoblots showing rhythms in oxidation of peroxiredoxin (PRDX-SO_2/3_ dimer). Molecular weights are in kDa. Similarly, scans of Coomassie Blue stained gels (loading control) for each blot are shown alongside. The areas cropped with a bounding box are shown in Fig. S2.

Supplementary Figure 4

**
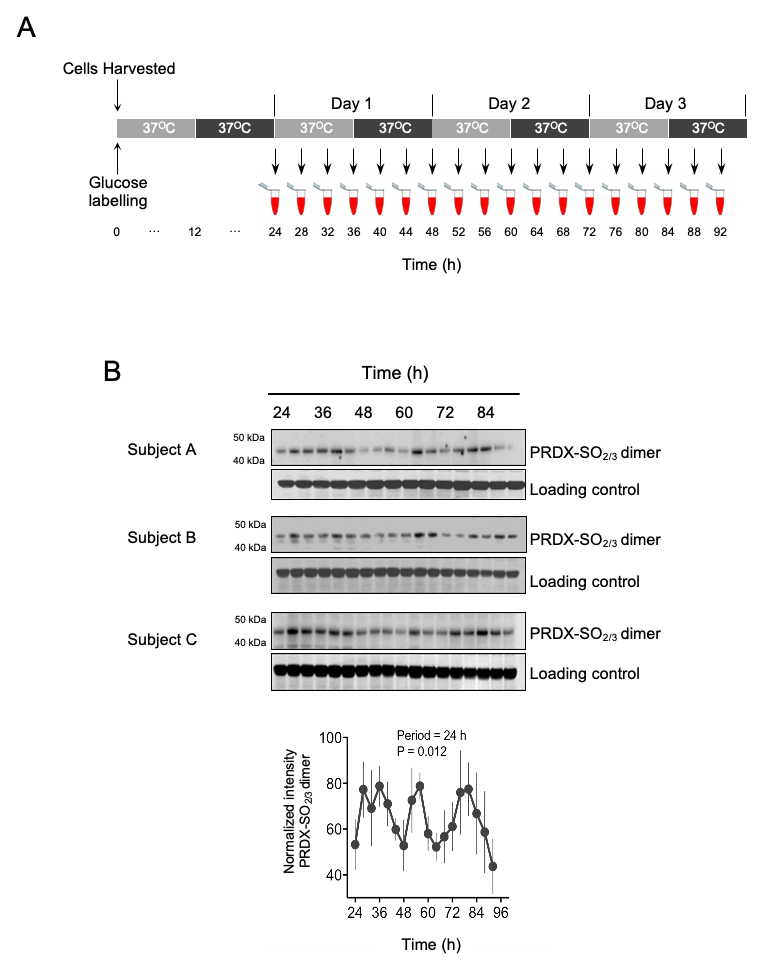
**

**Supplementary Figure 4. Peroxiredoxin oxidation rhythms in human red blood cells (RBCs) labeled with 1,2-^13^C_2_-Glucose (Related to Figure 3G)**

(A) Schematic showing experimental protocol used to collect samples. RBCs from n = 3 human subjects were incubated with 11mM 1,2-^13^C_2_-glucose and kept under constant conditions (37^o^C in continuous darkness) and sampling was performed every 4 hours after reaching steady state (see main text for details). (B) Immunoblots showing rhythms in oxidation of peroxiredoxin (PRDX-SO_2/3_) dimer with loading controls (Coomassie blue gel images). These blots correspond to flux labeling experiments shown in Fig 3E-F. Quantification of blots (mean ± s.e.m.) by densitometry is shown below (n = 3 biological replicates). Values are normalized to the maximum density within each blot. Uncropped blot images are shown in Fig. S5. P values were obtained from rhythmicity analysis using RAIN algorithm

Supplementary Figure 5

**
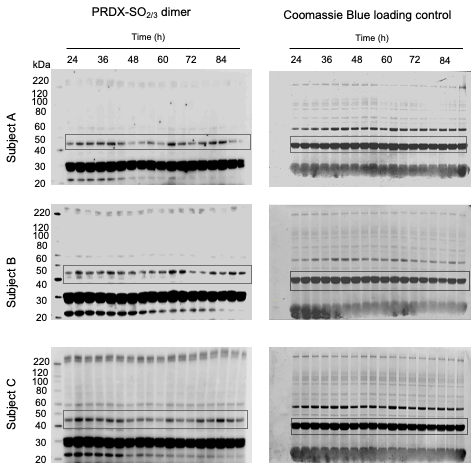
**

**Supplementary Figure 5. Circadian rhythms in oxidation of peroxiredoxin (PRDX-SO_2/3_ dimer) in human red blood cells (RBCs) (Related to Figure S4 and Figure 3G)**

Scans of whole immunoblots showing rhythms in oxidation of peroxiredoxin (PRDX-SO_2/3_ dimer). Molecular weights are in kDa. Similarly, scans of Coomassie Blue stained gels (loading control) for each blot are shown alongside. The areas cropped with a bounding box are shown in Fig. S4.

Supplementary Figure 6


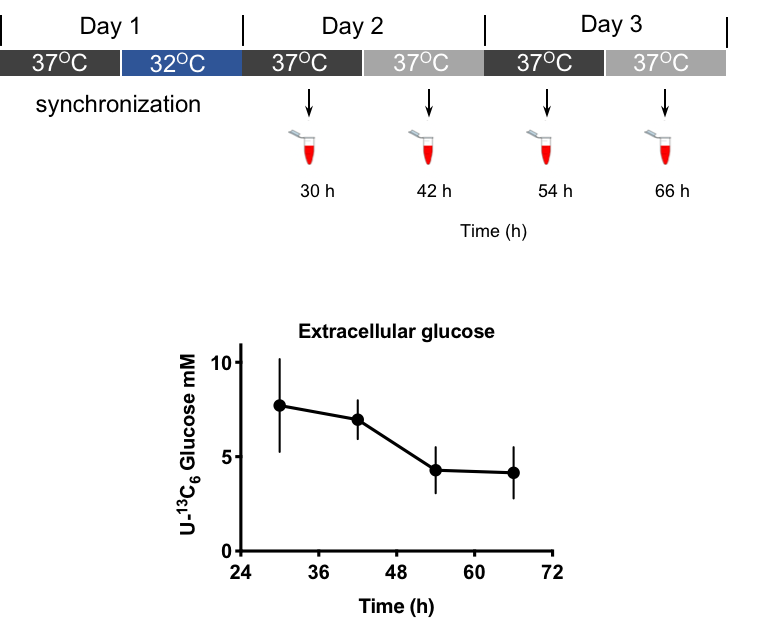


**Supplementary Figure 6. Glucose concentration in the extracellular medium of 13C-labelled glucose obtained from RBCs incubated with 11 mM of U-13C_6_-glucose.**

Samples were collected at the time-points shown over 48 hours as shown in the schematic. Glucose concentrations were measured by GC-MS (m/z 323 and retention time (Rt) 15.1 min). Data are mean±s.e.m. (n = 4 biological replicates).

Supplementary Figure 7


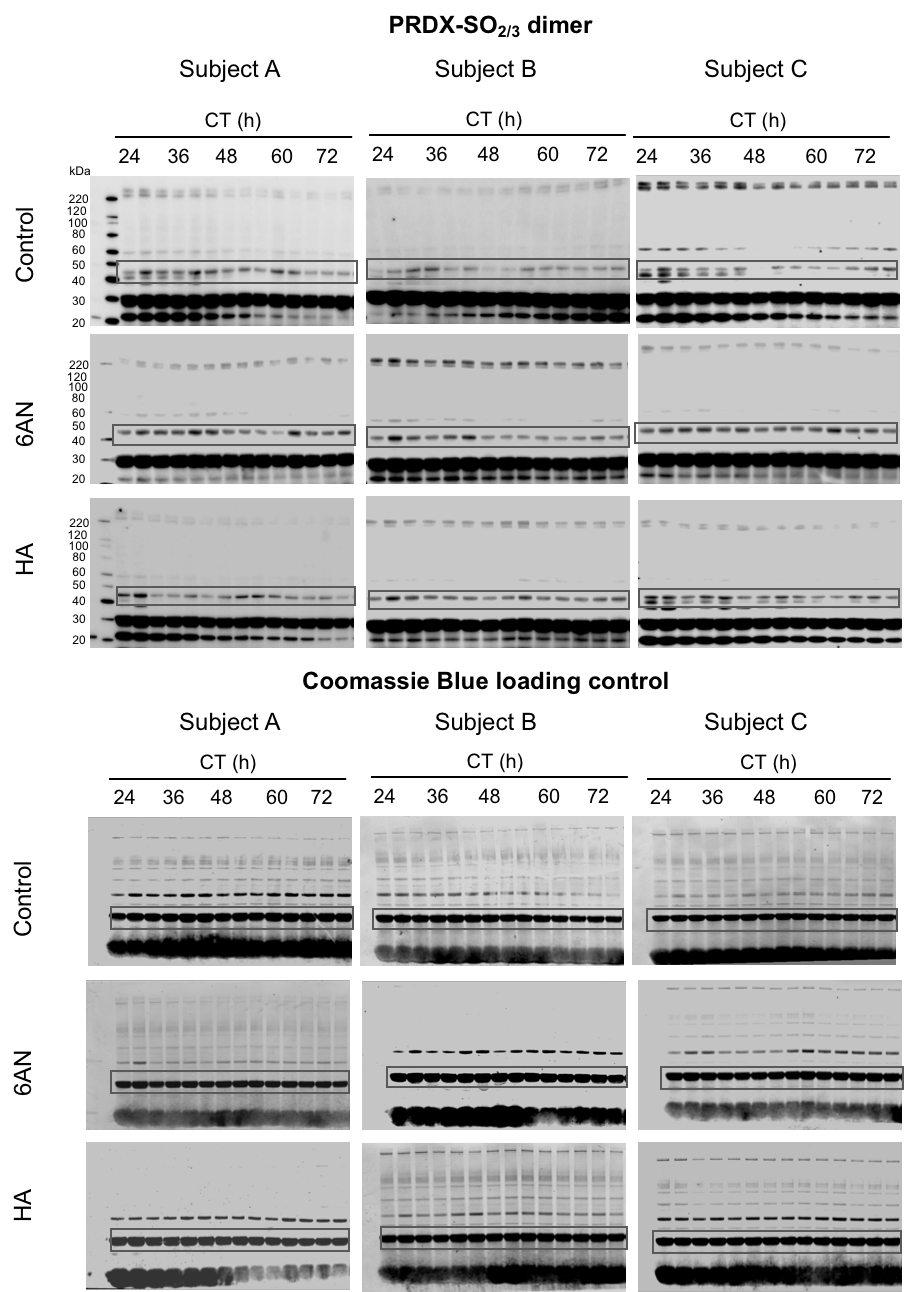


**Supplementary Figure 7. Circadian rhythms in oxidation of peroxiredoxin (PRDX-SO_2/3_ dimer) in human red blood cells (RBCs) treated with metabolic inhibitors (Related to Figure 5D)**

Scans of whole immunoblots showing rhythms in oxidation of peroxiredoxin (PRDX-SO_2/3_ dimer). Similarly, scans of Coomassie Blue stained gels (loading control) for each blot are shown below. Molecular weights are in kDa. Quantification of the blots corresponds to data in Fig. 5D.

Supplementary Figure 8


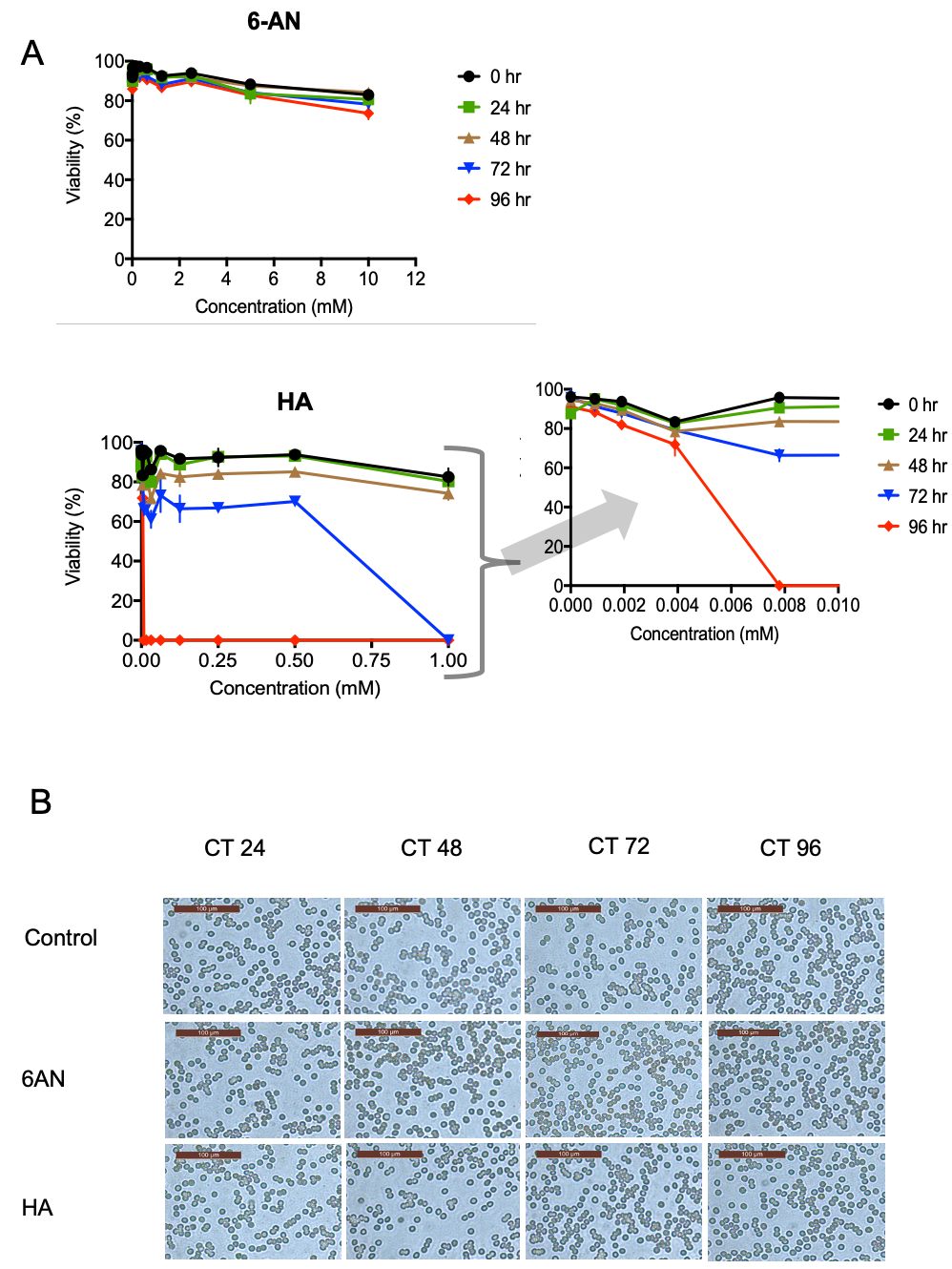


**Supplementary Figure 8. Assessment of cell viability of human red blood cells (RBCs) treated with metabolic inhibitors (Related to Figure 5 and Figure 6)**

(A) RBCs were treated with 6-Aminonicotinamide (6AN) or heptelidic acid (HA) at a range of concentrations, and cell viability assessed by quantifying the degree of RBC lysis (see Methods). Profiles are shown at t = 0, 24, 48, 72 and 96 h after adding the drug at each concentration. Data are mean ± s.e.m. (n = 3 biological replicates). The inset for heptelidic acid shows viability at low concentrations (0-8 µM), since RBC lysis started to become significant above this range. For 6AN, no significant difference in viability was seen at the highest concentration (10 mM), and therefore this dose was used for the main time course experiment. For HA, the threshold for normal viability at 96 h (> 80%) was at 1.9 µM, which was therefore used as the concentration in the main time course. (B) Representative brightfield microscope images of RBCs during time course with metabolic inhibitors and control. No morphological changes (e.g. crenation or cell size) were observed in any field of view. Scale bar represents 100 µm.

Supplementary Figure 9


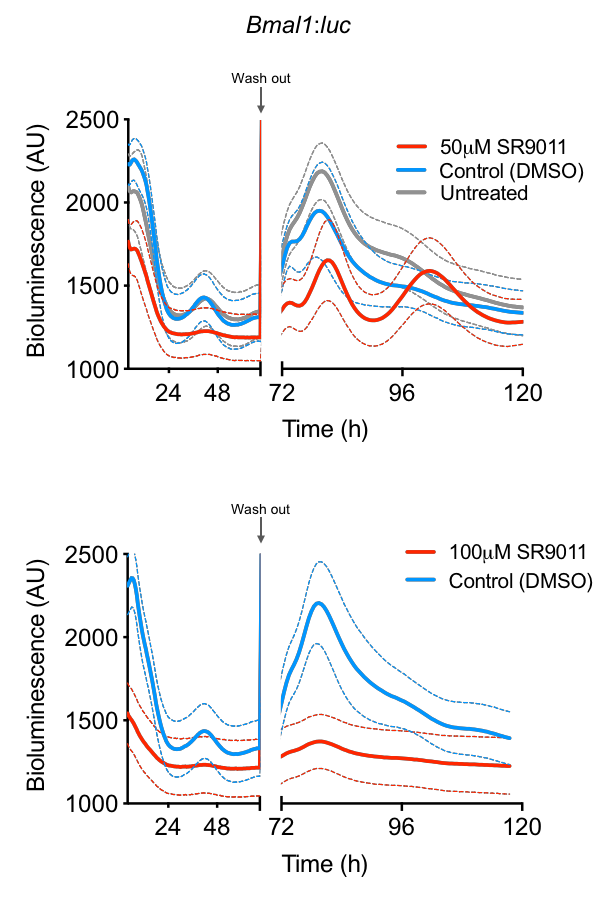


**Supplementary Figure 9. Damping of transcriptional circadian rhythms by REV-ERB agonist SR9011 (Related to Figure 7C and 7D)**

Treatment with 50 µM SR9011, a REV-ERB agonist, resulted in severe damping of bioluminescence rhythms that was reversible on drug wash out (shown by arrow). Traces for cells treated with control (DMSO) are also shown. Treatment with 100 µM SR9011 led to damped rhythms acutely, but rhythms were not apparent when the drug was washed out. Data are mean ± s.e.m. (n = 4 biological replicates; error boundaries shown by dashed lines).

Supplementary Figure 10


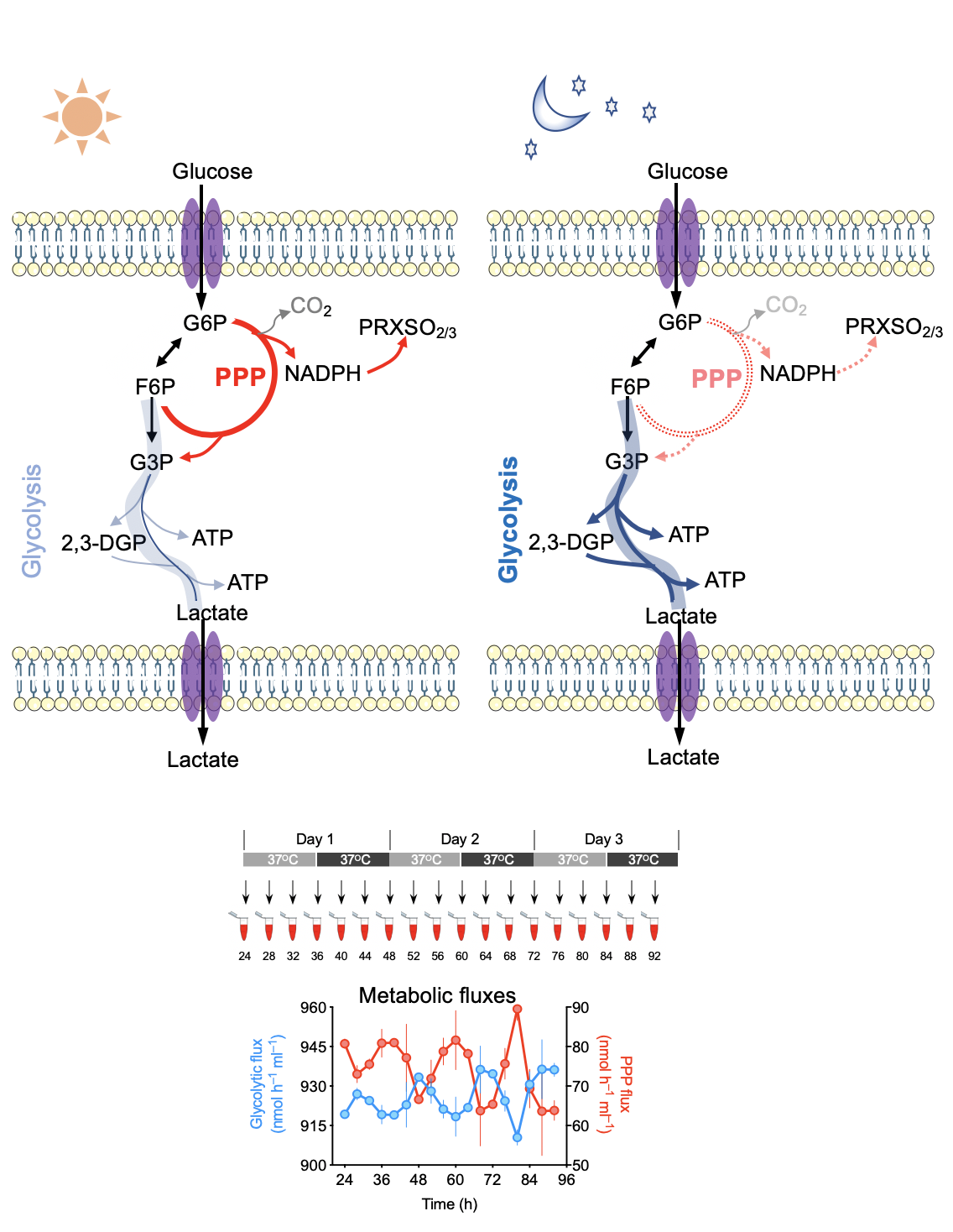


**Supplementary Figure 10. Model of red blood cell glucose metabolism over the circadian day and night**

In the circadian daytime, pentose phosphate pathway (PPP) flux reaches its peak whereas glycolysis is at its nadir. Reciprocally, at night, glycolytic flux peaks as PPP flux reaches its lowest level. G6P = Glucose-6-phosphate, F6P = Fructose-6-phosphate, ATP = Adenosine triphosphate, 2,3-BPG = 2,3-Biphosphoglycerate, G3P = Glyceraldehyde-3-phosphate, NADPH = Nicotinamide adenine dinucleotide phosphate reduced form, PRDX-SO_2/3_ = overoxidized peroxiredoxin protein. The lower panel of the figure is obtained from fig 3C Graph bars present mean ± s.e.m (n=3 biological replicates).

Supplementary Table 1

**Supplementary Table 1** Identified rhythmic metabolites in Human red blood cells analyzed by LC/GC-MS

| Metabolite | m/z | Rt | Platform | HMDB | KEGGID |
| --- | --- | --- | --- | --- | --- |
| Glucose | 319.00 | 15.27 | GC-MS | 00122 | C00031 |
| 3-Phosohoglycerate | 357.00 | 14.31 | GC-MS | 00807 | C00197 |
| Glucose-6-Phosphate | 387.00 | 18.19 | GC-MS | 01401 | C00668 |
| Serine | 204.00 | 10.02 | GC-MS | 00187 | C00065 |
| Ribulose-5Phosphate | 315.00 | 16.73 | GC-MS | 00618 | C00199 |
| Aminomalonicacid | 320.00 | 11.18 | GC-MS | 01147 | C00872 |
| Proline | 142.00 | 9.40 | GC-MS | 00162 | C00148 |
| Sedoheptulose-7-Phosphate | 387.00 | 19.8 | GC-MS | 01068 | C05382 |
| Isoleucine | 158.00 | 9.32 | GC-MS | 00172 | C00407 |
| Phenylalanine | 218.00 | 12.75 | GC-MS | 00159 | C00079 |
| Valine | 144.00 | 8.47 | GC-MS | 00883 | C00183 |
| Pyruvate | 87.0083 | 9.16 | LC-MS neg | 00243 | C00022 |
| Lactate | 89.024 | 10.25 | LC-MS neg | 00190 | C00186 |
| Glycolic acid | 75.0082 | 5.96 | LC-MS neg | 00115 | C00160 |
| Uracil | 111.0199 | 9.69 | LC-MS neg | 00296 | C00106 |
| Aspartic acid | 114.0195 | 7.39 | LC-MS neg | 00191 | C00049 |
| Nicotinamide | 121.0405 | 6.94 | LC-MS neg | 01406 | C00153 |
| Glutamine | 127.0511 | 12.44 | LC-MS neg | 00641 | C00064 |
| Creatine | 130.062 | 12.42 | LC-MS neg | 00064 | C00300 |
| Glutathione reduced | 308.091 | 12.27 | LC-MS pos | 00125 | C00051 |
| Gluconic acid | 175.0245 | 14.03 | LC-MS neg | 00625 | C00257 |
| 2-phosphoglyceric acid | 184.9853 | 13.19 | LC-MS neg | 00362 | C00631 |
| 2,3-Diphosphoglycerate cyclic | 268.924 | 20.48 | LC-MS neg | 01294 | C01159 |
| Ribose-5-Phosphate | 229.0114 | 12.79 | LC-MS neg | 01548 | C00117 |
| Uridine | 243.0618 | 9.69 | LC-MS neg | 00296 | C00299 |
| 6-phospho-2-dehydro-D-gluconate | 273.0014 | 12.97 | LC-MS neg | - | C01218 |
| 6-phosphogluconicacid | 275.0168 | 13.5 | LC-MS neg | 01316 | C00345 |
| Glutathione Oxidized | 305.0682 | 13.13 | LC-MS neg | 03337 | C00127 |
| Fructose-1,6-biphosphate | 338.988 | 13.25 | LC-MS neg | 01058 | C05378 |
| ADP | 426.0215 | 12.2 | LC-MS neg | 01341 | C00008 |
| ATP | 505.9874 | 12.75 | LC-MS neg | 00538 | C00002 |
| NAD+ | 664.1157 | 11.81 | LC-MS pos | 00902 | C00003 |
| Threonine | 120.0657 | 12.44 | LC-MS pos | 00167 | C00188 |
| Leucine | 132.1021 | 11.69 | LC-MS pos | 00687 | C00123 |
| 1,3-Diphosphoglycerate | 133.9867 | 9.15 | LC-MS pos | 01270 | C00236 |
| Arginine | 175.1192 | 16.72 | LC-MS pos | 00517 | C00062 |
| Citrulline | 176.1031 | 13.57 | LC-MS pos | 00904 | C00327 |
| Sorbitol | 183.0846 | 11.75 | LC-MS pos | 00247 | C00794 |
| UDP | 203.0067 | 14.14 | LC-MS pos | 00295 | C00015 |
| Heme | 309.0945 | 12.82 | LC-MS pos | 03178 | C00032 |
| Ornithine | 133.0972 | 15.17 | LC-MS pos | 00214 | C00077 |
| Asparaginylhydroxyproline | 246.1085 | 11.71 | LC-MS pos | 28732 | - |
| Leucyl-Proline | 229.1546 | 6.93 | LC-MS pos | 11175 | - |
