# Supplementary material for: Rhythmic glucose metabolism regulates the redox circadian clockwork in human red blood cells
Source: Nat Commun. 2021 Jan 15;12:377. doi: 10.1038/s41467-020-20479-4 (PMC7810875; doi:10.1038/s41467-020-20479-4)
Supplement: Supplementary file 3 — Reporting Summary [file 41467_2020_20479_MOESM3_ESM.pdf]

nature research

Corresponding author(s): Akhilesh B. Reddy

Last updated by author(s): Oct 20, 2020

Reporting Summary

Nature Research wishes to improve the reproducibility of the work that we publish. This form provides structure for consistency and transparency in reporting. For further information on Nature Research policies, see our Editorial Policies and the Editorial Policy Checklist.

Statistics

For all statistical analyses, confirm that the following items are present in the figure legend, table legend, main text, or Methods section.

n/a	Confirmed	
☐	☒	The exact sample size (n) for each experimental group/condition, given as a discrete number and unit of measurement
☐	☒	A statement on whether measurements were taken from distinct samples or whether the same sample was measured repeatedly
☐	☒	The statistical test(s) used AND whether they are one- or two-sided<br>*Only common tests should be described solely by name; describe more complex techniques in the Methods section.*
☐	☒	A description of all covariates tested
☐	☒	A description of any assumptions or corrections, such as tests of normality and adjustment for multiple comparisons
☐	☒	A full description of the statistical parameters including central tendency (e.g. means) or other basic estimates (e.g. regression coefficient) AND variation (e.g. standard deviation) or associated estimates of uncertainty (e.g. confidence intervals)
☐	☒	For null hypothesis testing, the test statistic (e.g. F, t, r) with confidence intervals, effect sizes, degrees of freedom and P value noted<br>*Give P values as exact values whenever suitable.*
☒	☐	For Bayesian analysis, information on the choice of priors and Markov chain Monte Carlo settings
☐	☒	For hierarchical and complex designs, identification of the appropriate level for tests and full reporting of outcomes
☐	☒	Estimates of effect sizes (e.g. Cohen's d, Pearson's r), indicating how they were calculated

Our web collection on statistics for biologists contains articles on many of the points above.

Software and code

Policy information about availability of computer code

Data collection	LC-MS data was acquired using Xcalibur 3.0.63 software (Thermo Scientific).<br>LC-MS data was acquired using MassHunter (Agilent QTOF) v B.08.00<br>Data were processed and integrated using Waters TargetLynx software (version 4.1) for 1,2-13C2 pulse labelling experiments<br>GC-MS data was acquired using MassHunter WorkStation (Agilent) B.07.00.<br>Immunoblot images were acquired using Amersham Imager 600 software.<br>Loading gel images were acquired using Odyssey version 3.0 from LI-COR.<br>RBC microscopy images were acquired using Leica LAS software version 4.8.<br>Red cell absorbance measurements were acquired using Tecan M1000 plate reader software.<br>NADH/NADPH absorbance measurements were acquired using Kaleido pate reader software.<br>Details are mentioned in the respective paragraphs in the Methods section.
Data analysis	Agilent Mass Hunter B.07.00 (GC-MS), Xcalibur 3.0.63 (LC-MS), Progenesis QI (LC-MS), Agilent MassHunter Profinder v B.08.00 (Batch Isotopologue extraction), IsoCor v1(isotope correction), Topspin 3.5 (NMR), NIH Image J 1.50 (blot quantification), GraphPad Prism v7 & 8, R v3.4 and R packages (CellulaRhythm and Metabanalyst software v 4.0) were used in analysis. Details are mentioned in the respective paragraphs in the Methods section.

For manuscripts utilizing custom algorithms or software that are central to the research but not yet described in published literature, software must be made available to editors and reviewers. We strongly encourage code deposition in a community repository (e.g. GitHub). See the Nature Research guidelines for submitting code & software for further information.

Data

Policy information about availability of data

All manuscripts must include a data availability statement. This statement should provide the following information, where applicable:

- Accession codes, unique identifiers, or web links for publicly available datasets
- A list of figures that have associated raw data
- A description of any restrictions on data availability

All data are available from the authors upon request. Source Data are provided with the online version of the paper: for Fig. 3D it is provided in Figure S2 & Figure S3, that for Figure 3G in Figure. S4 and Figure S5, that for Figure 5D in Figure 5 & 6 in Figure S8, and that for Figure 7C & 7D in Figure S9. Untargeted metabolomics data for Fig 1. are available on the Metabolights platform. Study submission code: MTBLS1285 and link https://www.ebi.ac.uk/metabolights/MTBLS1285

Field-specific reporting

Please select the one below that is the best fit for your research. If you are not sure, read the appropriate sections before making your selection.

[x] Life sciences   [ ] Behavioural & social sciences   [ ] Ecological, evolutionary & environmental sciences

For a reference copy of the document with all sections, see nature.com/documents/nr-reporting-summary-flat.pdf

Life sciences study design

All studies must disclose on these points even when the disclosure is negative.

Sample size	No statistical methods were used to predetermine sample size. Sample sizes were determined based on measured variations of interested quantities.
Data exclusions	No data were excluded from the analyses.
Replication	For all experiments n = 3 to 4 biological replicates were used. All attempts at replication were successful.
Randomization	Human samples collected from subjects are randomly allocated to experimental groups.
Blinding	Investigators were not blinded to sample identity - not relevant to this study and impossible to implement practically given the number of time points / samples / conditions used, and also to ensure that batches of samples were run in on instruments at the same time (to avoid batch effects).

Reporting for specific materials, systems and methods

We require information from authors about some types of materials, experimental systems and methods used in many studies. Here, indicate whether each material, system or method listed is relevant to your study. If you are not sure if a list item applies to your research, read the appropriate section before selecting a response.

Materials & experimental systems

n/a	Involved in the study	
[ ]	[x]	Antibodies
[ ]	[x]	Eukaryotic cell lines
[x]	[ ]	Palaeontology and archaeology
[ ]	[x]	Animals and other organisms
[ ]	[x]	Human research participants
[x]	[ ]	Clinical data
[x]	[ ]	Dual use research of concern

Methods

n/a	Involved in the study	
[x]	[ ]	ChIP-seq
[x]	[ ]	Flow cytometry
[x]	[ ]	MRI-based neuroimaging

Antibodies

Antibodies used	Anti-Peroxiredoxin-SO3, Abcam ab16830, (Lot no GR3202756, GR305645), Anti-Rabbit IgG (whole molecule) peroxidase conjugate Sigma A-6154 (Lot no 90K9175).
Validation	Anti-Peroxiredoxin-SO3, Abcam ab16830, (Lot no GR3202756, GR305645), Anti-Rabbit IgG (whole molecule) peroxidase conjugate Sigma A-6154 (Lot no 90K9175). Validated in WB, IHC and tested in Mouse, Rat, Human.<br>1. O'Neill JS & Reddy AB Circadian clocks in human red blood cells. Nature 469:498-503 (2011). WB ; Human<br>2. Edgar RS et al. Peroxiredoxins are conserved markers of circadian rhythms. Nature 485:459-64 (2012). WB ; Mouse,Drosophila melanogaster,Neurospora crassa .<br>3. Talaei F et al. Increased protein aggregation in Zucker diabetic fatty rat brain: identification of key mechanistic targets and the

therapeutic application of hydrogen sulfide. BMC Cell Biol 15:1 (2014). WB ; Rat .

Eukaryotic cell lines

Policy information about cell lines

Cell line source(s)	Bmal1:luc U2OS cells were a kind gift of Dr Andrew Liu (University of Memphis) (described in Liu et al., PLoS Genet. 4, e1000023 (2008)). Bmal1 KO and WT fibroblasts-generated by our lab.
Authentication	PCR genotyping for Bmal1 KO and WT cells. Bmal1:luc U2OS cells were not authenticated.
Mycoplasma contamination	All cell lines were tested and found negative for Mycoplasma contamination.
Commonly misidentified lines (See ICLAC register)	No commonly misidentified cell lines were used.

Animals and other organisms

Policy information about studies involving animals; ARRIVE guidelines recommended for reporting animal research

Laboratory animals	Male C57BL/6J mice, 8-10 weeks old, were purchased from Charles River
Wild animals	Study did not involve Wild animals
Field-collected samples	Study did not involve samples collected from the field
Ethics oversight	Animal experiments were performed under license by the Home Office-the Animals (Scientific Procedures) Act 1986, with Local Ethical Review by the Francis Crick Institute Animal Welfare & Ethical Review Body Standing Committee (AWERB) or in concordance with an approved protocol from Institutional Animal Care and Use Committee (IACUC) at Perelman School of Medicine at the University of Pennsylvania

Note that full information on the approval of the study protocol must also be provided in the manuscript.

Human research participants

Policy information about studies involving human research participants

Population characteristics	Participants were healthy adults (18-67 years of age) and there was no gender specification.
Recruitment	All volunteers provided written informed consent after receiving a participation information sheet containing detailed information of the study procedures. All volunteers provided written informed consent after receiving a participation information sheet containing detailed information of the study procedures.
Ethics oversight	Studies were conducted with approval from the Health Research Authority’s (UK) Research Ethics Committee (Reference number 12/EE/0370) and local ethical approval by The Francis Crick Institute’s Ethics Review Board. The Francis Crick Institute is licensed by the Human Tissue Authority (HTA) to store human samples for the purposes of research (Licence number 12650). The research complies with all requirements of the relevant HTA Code of Practice.

Note that full information on the approval of the study protocol must also be provided in the manuscript.
